# On a boundary property of analytic functions

**DOI:** 10.1186/s13660-017-1575-9

**Published:** 2017-11-28

**Authors:** Mamoru Nunokawa, Janusz Sokół

**Affiliations:** 1 University of Gunma, Hoshikuki-cho 798-8, Chuou-Ward, Chiba, 260-0808 Japan; 2 Faculty of Mathematics and Natural Sciences, University of Rzeszów, ul. Prof. Pigonia 1, Rzeszów, 35-310 Poland

**Keywords:** 30C45, 30C80, analytic functions, meromorphic functions, univalent functions, boundary behavior

## Abstract

Let *f* be an analytic function in the unit disc $|z|<1$ on the complex plane $\mathbb {C}$. This paper is devoted to obtaining the correspondence between $f(z)$ and $zf'(z)$ at the point *w*, $0<|w|=R< 1$, such that $|f(w)|=\min \{|f(z)|: f(z)\in\partial f(|z|\leq R) \}$. We present several applications of the main result. A part of them improve the previous results of this type.

## Introduction

Let ${\mathcal {H}}$ denote the class of analytic functions in the unit disc $|z|<1$ on the complex plane ${\mathbb{C}}$. The following lemma is a particular case of the Julia-Wolf theorem. It is known as Jack’s lemma.

### Lemma 1.1

([1])

*Let* $\omega(z)\in{\mathcal {H}}$ *with* $\omega(0)=0$. *If for a certain* $z_{0}$, $|z_{0}|<1$, *we have* $|\omega(z)|\leq|\omega(z_{0})|$ *for* $|z|\leq|z_{0}|$, *then* $z_{0}\omega'(z_{0})=m\omega(z_{0})$, $m\geq 1$.

In this paper, we consider a related problem. We establish a relation between $w(z)$ and $zw'(z)$ at the point $z_{0}$ such that $|w(z_{0})|=\min \{|w(z)|:|z|= |z_{0}| \}$ or at the point $z_{0}$ satisfying (1.1). We consider the *p*-valent functions.

### Lemma 1.2

*Let* $w(z) = z^{p} + \sum_{n=p+1} ^{\infty}a_{n} z^{n}$ *be analytic in* $|z| < 1$. *Assume that there exists a point* $z_{0} $, $|z_{0}|=R<1$, *such that*

1.1 $$ \min \bigl\{ \big|w(z)\big|: w(z)\in\partial w\bigl(|z|\leq R\bigr) \bigr\} = \big|w(z_{0})\big|>0. $$

*If* $w(z)/z^{p}\neq0$ *in* $|z| < R$, *then*

1.2 $$ \frac{z_{0}w'(z_{0})}{w(z_{0})}=k_{1}\leq p. $$

*If the function* $w(z)/z^{p}$ *has a zero in* $|z| < R$ *and* $\partial w(|z|\leq R)$ *is a smooth curve at* $w(z_{0})$, *then*

1.3 $$ \frac{z_{0}w'(z_{0})}{w(z_{0})}=k_{2}\geq p, $$

*where* $k_{1}$, $k_{2}$ *are real*.

### Proof

If

$$ \min \bigl\{ \big|w(z)\big|: w(z)\in\partial w\bigl(|z|\leq R\bigr) \bigr\} = \big|w(z_{0})\big|>0, $$

then

1.4 $$ \bigl\vert w(z) \bigr\vert \geq \bigl\vert w(z_{0}) \bigr\vert \quad\text{for } w(z)\in\partial w\bigl(|z|\leq R\bigr). $$

Then, we also have

1.5 $$ \biggl\vert \frac{w(z)}{z^{p}} \biggr\vert \geq \biggl\vert \frac{w(z_{0})}{z^{p}_{0}} \biggr\vert \quad\text{for } w(z)\in\partial w\bigl(|z|\leq R\bigr). $$

Let

1.6 $$ \Phi(z) = w(z)/z^{p}, \quad|z|< 1. $$

Then, from (1.5) and from hypothesis (1.1) we have

1.7 $$ \min \bigl\{ \big|\Phi(z)\big|:\Phi(z)\in\partial\Phi\bigl(|z|\leq R\bigr) \bigr\} = \big| \Phi(z_{0})\big|. $$

**Figure 1:**
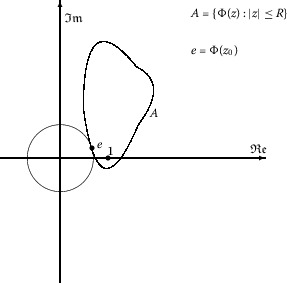
$\pmb{\Phi(z)}$ **in the case** $\pmb{\Phi(z)\neq0}$ **.**

**Figure 2:**
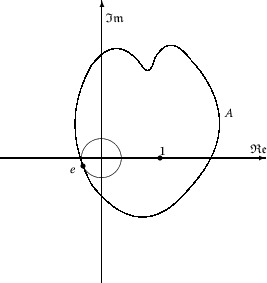
$\pmb{\Phi(z)}$ **in the case** $\pmb{0\in\Phi(|z|\leq R)}$ **.**

There are two cases: $\Phi(|z|< R)$ contains the origin (see Figure 2); and $\Phi(|z|< R)$ does not (see Figure 1).

First, suppose that $\Phi(z)$ does not become 0 in $|z| < R$. If there exists a point $z_{0}=R\exp(i\varphi_{0})$, $0\leq\varphi_{0}<2\pi$, $0 < R< 1$, such that

1.8 $$ \min \bigl\{ \big|\Phi(z)\big|: \Phi(z)\in\partial\Phi\bigl(|z|\leq R\bigr) \bigr\} = \big|\Phi(z_{0})\big|, $$

then the function

$$ F(z)=\frac{z}{\Phi(z)}=\frac{z^{p+1}}{w(z)}, \quad|z| \leq R, $$

satisfies the assumptions of Jack’s lemma (Lemma 1.1),

$$ F(z_{0})=\max_{\theta\in[0,2\pi)} \bigl\{ \big|F(z)\big|: z=Re^{i\theta} \bigr\} , $$

and hence

$$ \frac{z_{0}F'(z_{0})}{F(z_{0})}=p+1-\frac{z_{0}w'(z_{0})}{w(z_{0})}\geq m\geq1. $$

This gives (1.2).

For the case $0\in\Phi(|z|< R)$ (see Figure 2), for $\Phi(z)$ given in (1.6), we have that $|\Phi(z)|$ has an extremum at $z_{0}$, and so

1.9 $$ \frac{\mathrm{d}|\Phi(z)|}{\mathrm{d}\varphi} \bigg\vert _{z=z_{0}}=0. $$

Furthermore, $\arg \{\Phi(z) \}$ is increasing at $z_{0}$, and so

1.10 $$ \frac{\mathrm{d}\arg \{\Phi(z) \}}{\mathrm{d}\varphi} \bigg\vert _{z=z_{0}}\geq0. $$

Then we have

1.11 $$\begin{aligned}[b] \frac{z_{0}\Phi'(z_{0})}{\Phi(z_{0})} &= \frac{\mathrm{d}\log\Phi (z)}{\mathrm{d}\log z} \bigg\vert _{z=z_{0}} \\ &= \frac{\mathrm{d}\log|\Phi(z)|+i\mathrm{d}\arg \{\Phi(z) \} }{i\mathrm{d}\varphi} \bigg\vert _{z=z_{0}} \\ &= \frac{\mathrm{d}\arg \{\Phi(z) \}}{\mathrm{d}\varphi}- \frac{i}{|\Phi(z)|}\frac{\mathrm{d}|\Phi(z)|}{\mathrm{d}\varphi} \bigg\vert _{z=z_{0}} \\ &= \frac{\mathrm{d}\arg \{\Phi(z) \}}{\mathrm{d}\varphi} \bigg\vert _{z=z_{0}} \\ &\geq0,\end{aligned} $$

because of (1.9). On the other hand, by (1.6) we have $w'(z)=z^{p}\Phi'(z)+pz^{p-1}\Phi(z)$, and hence

1.12 $$ \frac{z_{0}w'(z_{0})}{w(z_{0})}=\frac{z_{0}\Phi'(z_{0})}{\Phi(z_{0})}+p. $$

Relations (1.11) and (1.12) imply that

$$ \frac{z_{0}w'(z_{0})}{w(z_{0})}\geq p. $$

Therefore, by (1.11) we obtain (1.3). □

If we additionally assume that $w(z)/z^{p}$ is univalent in the unit disc, then we have the following result.

### Remark 1.3

Let $w(z) = z^{p} + \sum_{n=p+1} ^{\infty}a_{n} z^{n}$ be analytic in $|z| < 1$. Assume that there exists a point $z_{0} $, $|z_{0}|=R<1$, such that

1.13 $$ \min_{\theta\in[0,2\pi)} \bigl\{ \big|w(z)\big|: z=Re^{i\theta} \bigr\} = \big|w(z_{0})\big|>0. $$

If $w(z)/z^{p}$ is univalent and $w(z)/z^{p}\neq0$ in $|z| \leq R$, then

1.14 $$ \frac{z_{0}w'(z_{0})}{w(z_{0})}=k_{1}\leq p, $$

where $k_{1}$ is real. If $w(z)/z^{p}$ is univalent and $w(z)/z^{p}$ vanishes in $|z| \leq R$, then

1.15 $$ \frac{z_{0}w'(z_{0})}{w(z_{0})}=k_{2}\geq p, $$

where $k_{2}$ is real.

## Applications

For $p=0$, then Lemma 1.2 becomes the following corollary.

### Corollary 2.1

*Let* $w(z) = 1 + \sum_{n=1} ^{\infty}a_{n} z^{n}$ *be analytic in* $|z| < 1$. *Assume that there exists a point* $z_{0} $, $|z_{0}|=R<1$, *such that*

2.1 $$ \min \bigl\{ \big|w(z)\big|: w(z)\in\partial w\bigl(|z|\leq R\bigr) \bigr\} = \big|w(z_{0})\big|>0. $$

*If* $w(z)\neq0$ *in* $|z| < R$, *then*

2.2 $$ \frac{z_{0}w'(z_{0})}{w(z_{0})}=k_{1}\leq0. $$

*If the function* $w(z)$ *has a zero in* $|z| < R$ *and* $\partial w(|z|\leq R)$ *is a smooth curve at* $w(z_{0})$, *then*

2.3 $$ \frac{z_{0}w'(z_{0})}{w(z_{0})}=k_{2}\geq0. $$

A simple contraposition of Lemma 1.2 provides the following two corollaries.

### Corollary 2.2

*Let* $w(z) = z^{p} + \sum_{n=p+1} ^{\infty}a_{n} z^{n}$ *be analytic in* $|z| < 1$ *and suppose that there exists a point* $z_{0} $, $|z_{0}|=R<1$, *such that*

2.4 $$ \min \bigl\{ \big|w(z)\big|: w(z)\in\partial w\bigl(|z|\leq R\bigr) \bigr\} = \big|w(z_{0})\big|>0. $$

*If*

2.5 $$ \frac{z_{0}w'(z_{0})}{w(z_{0})}=k_{1}< p $$

*and* $\partial w(|z|\leq R)$ *is a smooth curve at* $w(z_{0})$, *then* $w(z)/z^{p}$ *has no zero in* $|z| \leq R$. *If*

2.6 $$ \frac{z_{0}w'(z_{0})}{w(z_{0})}=k_{2}> p, $$

*then the function* $w(z)/z^{p}$ *has a zero in* $|z| \leq R$.

### Corollary 2.3

*Let* $q(z)=z^{p}+\sum_{n=p+1}^{\infty}a_{n} z^{n}$ *be analytic in* $|z|\leq1$. *Assume that* $q(z)/z^{p}$ *has a zero in* $|z|<1$. *If for given* $c\in[0,1)$,

2.7 $$ \big|zq'(z)\big|< \frac{p}{c}\big|q(z)\big|^{2},\quad |z| < 1, $$

*then the image domain* $q(|z|<1)$ *covers the disc* $|w|< c$.

### Proof

If

2.8 $$ \min \bigl\{ \big|q(z)\big|: q(z)\in\partial q\bigl(|z|\leq1\bigr) \bigr\} = \big|q(z_{0})\big|< c, $$

then by (1.2) in Lemma 1.2 we have

2.9 $$ \frac{z_{0}q'(z_{0})}{q(z_{0})}=k\geq p\quad \Rightarrow \quad\big|z_{0}q'(z_{0})\big| \geq p\big|q(z_{0})\big|. $$

Therefore, by (2.8) and (2.9) we have

$$ \big|z_{0}q'(z_{0})\big|\geq\frac{p}{c}\big|q(z_{0})\big|^{2}, $$

which contradicts hypothesis (2.7) and therefore completes the proof. □

### Theorem 2.4

*Let* $p(z)$ *be analytic in* $|z|<1$ *with* $p(z) \neq0$, $|p(0)|>c$, *in* $|z| < 1$ *and suppose that*

2.10 $$ \big|p(z) + zp'(z)\big|>c,\quad |z| < 1, $$

*where* $c>0$, *and that*

2.11 $$ \mathfrak{Re} \biggl\{ \frac{zp'(z)}{p(z)} \biggr\} >-2, \quad |z| < 1. $$

*Then we have*

2.12 $$ \big|p(z)\big|> c, \quad |z| < 1. $$

### Proof

If there exists a point $z_{0}$, $|z_{0}| < 1$, such that

2.13 $$ \big|p(z)\big| > c \quad\text{for } |z| < |z_{0}| $$

and $| p(z_{0}) | = c$, then $p(|z|\leq|z_{0}|)$ has the shape as in Figure 1 and $\mathrm{d}|p(z)|/\mathrm{d}\varphi$, $z=re^{i\varphi}$, vanishes at the point $z=z_{0}$. Therefore, we have

2.14 $$ \begin{aligned}[b]\frac{z_{0}p'(z_{0})}{p(z_{0})}&= \frac{\mathrm{d}\log p(z)}{\mathrm{d}\log z} \bigg\vert _{z=z_{0}} \\ &= \frac{\mathrm{d}\log|p(z)|+i\mathrm{d}\arg \{p(z) \} }{i\mathrm{d}\varphi} \bigg\vert _{z=z_{0}} \\ &= \frac{\mathrm{d}\arg \{p(z) \}}{\mathrm{d}\varphi}- \frac{i}{|p(z)|}\frac{\mathrm{d}|p(z)|}{\mathrm{d}\varphi} \bigg\vert _{z=z_{0}} \\ &= \frac{\mathrm{d}\arg \{p(z) \}}{\mathrm{d}\varphi} \bigg\vert _{z=z_{0}} \\ &\leq0.\end{aligned} $$

From (2.11) and (2.14) we have

$$ -2< \frac{z_{0}p'(z_{0})}{p(z_{0})}\leq0, $$

and hence

$$ 0\leq \biggl\vert 1 + \frac{z_{0}p'(z_{0})}{p(z_{0})} \biggr\vert \leq1. $$

Then it follows that

2.15 $$ \big|p(z_{0}) + z_{0}p'(z_{0})\big| =\big|p(z_{0})\big| \biggl\vert 1 + \frac{z_{0}p'(z_{0})}{p(z_{0})} \biggr\vert \leq\big| p(z_{0})\big| = c, $$

which contradicts hypothesis (2.10) and therefore completes the proof. □

For some other geometrical properties of analytic functions, we refer to the papers [2–4].

## Conclusion

In this paper, we have presented a correspondence between an analytic function $f(z)$ and $zf'(z)$ at the point *w*, $0<|w|=R< 1$, in the unit disc $|z|<1$ on the complex plane such that $|f(w)|=\min \{|f(z)|: f(z)\in\partial f(|z|\leq R) \}$.
